# Metachronous Periadrenal Fatty Tissue Metastasis from Contralateral Renal Cell Carcinoma

**DOI:** 10.1155/2013/206078

**Published:** 2013-03-05

**Authors:** Erdal Alkan, Oguz Ozkanli, Derya Balbay

**Affiliations:** Department of Urology, Memorial Sisli Hospital, Piyalepasa Bulvarı Okmeydanı, Sisli 34385, İstanbul, Turkey

## Abstract

Contralateral adrenal metastases from renal cell carcinomas are not commonly seen. To our knowledge, we are presenting the first case of extraadrenal metastasis in the English literature, from the contralateral RCC 6 months after radical nephrectomy. Patient was treated with robotic right adrenalectomy for continuous growing of a de novo right adrenal mass of 6 × 4 × 3 cm in diameter. Tissue between vena cava, renal capsule, and inferior diaphragmatic surface was removed en block. Pathological evaluation revealed renal cell carcinoma within the fatty tissue abutting the adrenal capsule from outside with negative surgical margins. Our experience dictates that removing adrenal tissue only after identifying the adrenal borders may sometimes result in insufficient tumor removal. Therefore, adrenal containing tissue within the anatomic boundaries should be removed en block, if surgical removal is planned for metachronous tumor metastasis in the treatment of renal cell carcinoma.

## 1. Introduction

Renal cell carcinoma (RCC) can metastasize to almost every organ, including the lungs (50% to 60%), liver (30% to 40%), bones (30% to 40%), and brain (5%) [1]. Adrenal metastases are frequently found at autopsy, rarely metachronously after nephrectomy [2]. No metachronous contralateral periadrenal fatty tissue metastases from primary RCC have been reported. Herein, we present a patient with metachronous solitary metastasis of RCC to the contra lateral peri-adrenal fatty tissue treated with robotic adrenalectomy.

## 2. Case Report

**Figure 1:**
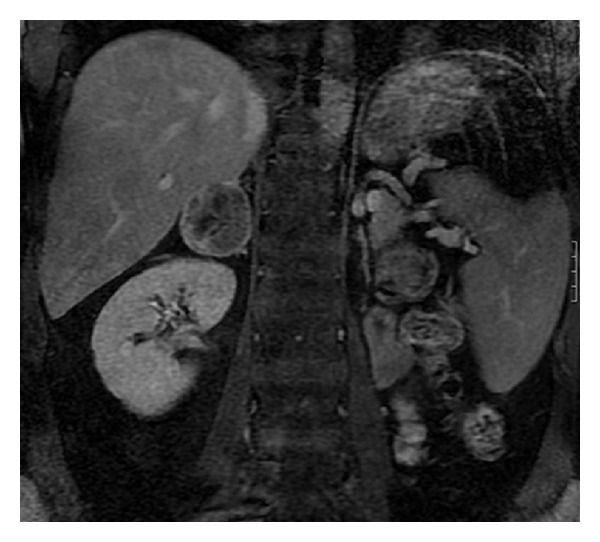
Preoperative magnetic resonance imaging (MRI) of right peri-adrenal tumor.

A 44-year-old male patient was detected with a right adrenal mass on this followup examination after left radical nephrectomy done for RCC. The pathology report of the nephrectomy specimen showed a Fuhrman grade 3 stage T2 (T2N0M0) RCC. At the postoperative 6 months of follow up, abdominal CT revealed a 6∗5 cm solid mass in the right adrenal. An MRI scan was obtained prior to surgery for better delineation of the right adrenal (Figure 1). Chest CT and bone scintigraphy demonstrated no abnormal findings. Laboratory tests were all within normal limits. In particular, metabolic evaluation included 24-hour urine collections for metanephrines, cortisol, 17-ketosteroids, 17-hydrocorticoids, and vanillylmandelic acid, which were within normal limits as well as physical examination findings. Based on these findings, the patient was scheduled for robotic adrenalectomy.

 We preferred a transperitoneal lateral decubitus approach as the best for maximal exposure of the adrenal gland and major vessels. Whole mass was dissected off of the neighboring structures including vena cava, liver, and the kidney without exposing the adrenal gland. The procedure lasted 2 hours, the estimated intraoperative blood loss was 350 mL, and the patient did not receive any transfusion.

**Figure 2:**
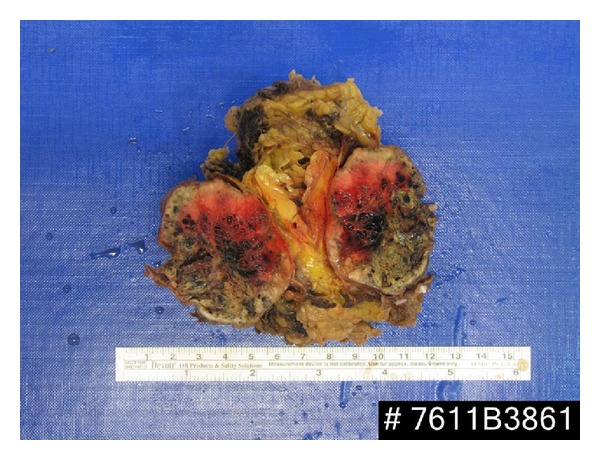
Gross pathologic evaluation of the resected specimen. The tumor was located in the peri-adrenal fatty tissue outside the adrenal gland.

**Figure 3:**
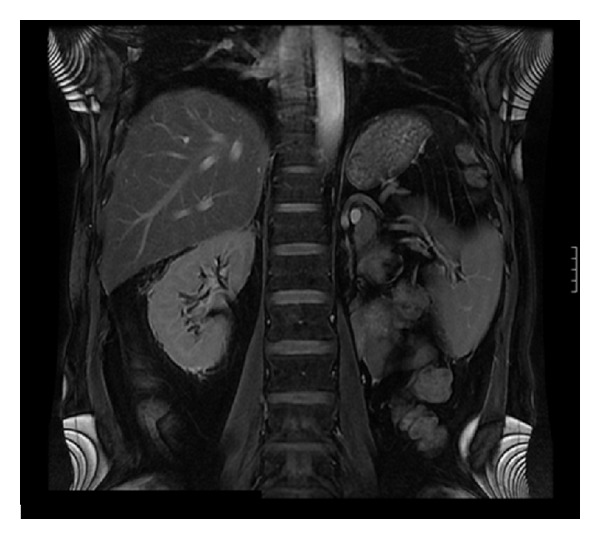
Postoperative magnetic resonance imaging (MRI) of the patient (6 months of follow up).

 The postoperative period was uneventful, and the patient was discharged on postoperative day 2. The histological examination of the specimen demonstrated a metastatic RCC with features similar to the primary renal tumor. Interestingly, the tumor was located in the periadrenal fatty tissue outside the adrenal gland abutting the adrenal capsule without any adrenal parenchymal invasion. All surgical margins of the resected mass were free of tumor. The specimen weighed 62 g and measured 6 × 4 × 3 cm (Figure 2). On 6 months of follow, up the patient was well without any evidence of metastasis as per radiologic evaluation (Figure 3). Since his peri-adrenal mass is a systemic metastasis, he was placed on tyrosine kinase inhibitor treatment.

## 3. Discussion

 Approximately 25% of patients with RCC will already have multiple distant metastases at the time of presentation, including the lungs, lymph nodes, liver, or bones [1]. Adrenal metastases from RCC are not uncommon; in nephrectomised patients, the incidence of solitary adrenal metastasis is 3% to the ipsilateral gland and only 0.7% to the contra lateral gland [3]. In reviews of the literature, the majority of the cases described consisted of synchronous metastasis [4]. Most metachronous metastases are identified in the first or second year after nephrectomy, and 25% of patients develop metastatic disease within 5 years of nephrectomy [5]. In our case, contra lateral peri-adrenal fatty tissue metastasis was detected after 6 months of nephrectomy. To our knowledge, such metastasis from RCC has never been reported in the literature yet.

Adrenal metastases are usually anatomically and functionally silent, and patients rarely have symptoms or signs of adrenal insufficiency. Thus, if abdominal imaging is not used routinely during the follow up, an isolated adrenal metastasis from RCC could be missed [6]. A solitary metastasis to the contra lateral adrenal gland can cause confusion, particularly since the histological status maybe unclear [7]. The optimal diagnostic approach to a solitary contra lateral adrenal tumor in patients with a history of RCC is contentious and seems to differ from the management of “incidentalomas” [8]. Radiological studies may facilitate the preoperative diagnosis but cannot determine with certainty whether an adrenal tumor in a patient with RCC is a primary adrenal neoplasm, an adrenal cortical adenoma, or a metastasis [8]. In our case, the normal metabolic screen and the recent history of RCC suggested adrenal metastasis as the most likely diagnosis.

The prognosis for patients with untreated metastatic renal cell carcinoma is dismal. Surgical removal is the only known effective treatment in patients with solitary adrenal metastasis, with 29% to 35% of them surviving 5 years or more [9]. Plawner showed that the 5-year survival of patients operated on for metachronous solitary RCC metastases to the contra lateral adrenal gland was lower than that for patients with synchronous adrenal metastases (20% and 40%, resp.) [9]. It has also been noted that patients diagnosed with adrenal metastasis a long time after nephrectomy had a better prognosis than those with a short interval to diagnosis. Among patients who undergo nephrectomy and resection of solitary metastases, it has been reported that 30% have prolonged survival, many of them for more than 5 years. Therefore, aggressive treatment-excision of such lesions is indicated [9].

Robotic adrenalectomy has been performed successfully to treat a variety of benign adrenal pathological conditions and is associated with less postoperative discomfort, decreased hospital stay, less postoperative disability, and a lower rate of complications. In our case, the metastatic mass measured 6 × 5 cm in preoperative imaging and was resected successfully through a robot-assisted transperitoneal approach, in a patient with a previous history of open radical nephrectomy of the contra lateral kidney. We want to punctuate one more time that-so-called adrenal masses should be removed without exposing the adrenal gland but instead dissecting the whole mass en block off of the surrounding tissue such as major vascular structures and neighboring organs.

## 4. Conclusion

To our knowledge, we are the first to report a case of metachronous, contralateral peri-adrenal fatty tissue metastasis from RCC. Our believe is that-so-called adrenal masses should be removed without exposing the adrenal gland but instead dissecting the whole mass enblock off of the surrounding tissue such as major vascular structures and neighboring organs.
